# Aiming for survival: a qualitative single case study of support for family members across the care process in outpatient colorectal cancer care

**DOI:** 10.1186/s12885-025-14245-2

**Published:** 2025-05-12

**Authors:** Maria Samuelsson, Kristina Edman, Merita Neziraj, Anna Ericsson

**Affiliations:** 1 Faculty of Health and Society, Department of Care Science, Malmö University, Jan Waldenströms gata 25, Malmö, 205 06 Sweden; 2 HØKH Department of Health Services Research, Akershus University Hospital, Nordbyhagen, Norway

**Keywords:** Cancer specialist nurses, Clinical social workers, Cancer, Family members, Qualitative case study, Psychosocial support

## Abstract

**Background:**

At times of cancer, also family members may need support from healthcare professionals. For support to be relevant it needs to be tailored to a person’s needs. Tailored support is recognized as support co-created through an intangible interaction between the supporter and the supported. Despite this, studies primarily focus on the supporter (healthcare professionals) or the supported (family members). As a result, the co-created dimension is lost. Therefore, the purpose was to describe and compare family members´ supportive care needs with support from cancer nurses across the care process in outpatient colorectal cancer care.

**Methods:**

This study is designed as a qualitative single case study with two embedded units: family members and Contact Nurses. Data consisted of transcribed semi-structured interviews from 23 family members and 21 Contact Nurses. Both within and across units, analyses were undertaken using conventional qualitative content analysis. Reporting adheres to the Consolidated Criteria for Reporting Qualitative Research checklist.

**Results:**

Analysis generated a main category: *Aiming for survival* illustrating the common goal of the two units and its implications for support for family members in routine colorectal cancer care. Three subcategories describe family members´ supportive care needs in relation to Contact Nurses´ support for family members across the colorectal cancer care process: (1) *The diagnostic phase: Narrowed sight in treatment preparation*; (2) *The treatment phase: Foregrounding family caregiving while backgrounding family support*; and (3) *The surveillance phase: An enduring cancer experience despite being considered a co-survivor.*

**Conclusions:**

Support tailored to family members’ supportive care needs should derive from the family members’ cancer experiences and include strategies for bringing their needs to light. This could possibly be achieved by strengthening the collaboration between contact nurses and clinical social workers. In addition, family members require preparation for and support during their entire cancer trajectory to enable a healthy family recovery post-treatment. In addition, they need guidance on where and whom to turn to at each stage of the care process.

**Supplementary Information:**

The online version contains supplementary material available at 10.1186/s12885-025-14245-2.

## Background

Cancer is a family affair [1]. Each phase of the cancer trajectory, from diagnosis to treatment and survival, or end of life, impacts the family members emotionally, existentially, and practically [2–4] and may result in negative health outcomes among the family members [2, 3, 5, 6]. As a result, support for family members has been recommended for decades [7–11], and multiple support interventions have been developed [7–12]. Despite extensive efforts, family members continue to report unmet supportive care needs [3, 13–15] and high rates of ill health [5, 14]. Suggested reasons are that support interventions are unimplementable in real-world settings or not tailored to family members’ individual needs [9, 12, 16, 17]. This motivates a continued study of how to support these family members in a way that is tailored to their needs yet implementable in routine cancer care. This study is part of a project aiming to develop a family support model for outpatient cancer care.

Due to the transformation of in-hospital cancer care to outpatient cancer care, the outpatient setting is recommended for supportive initiatives for family members [18–20]. Cancer specialist nurses employed at these clinics, for example, Contact Nurses (CNs), are highlighted as having a key position in supporting patients and family members across the care process. A *CN* is a registered nurse with expertise in specific cancer care and treatment [21]. *Family members* are “who they say they are” [22] — biological family members, next of kin, and/or friends. Given that studies have shown that the needs of family members and patients [15] as well as the needs of adults and children [23] may differ, this study focuses explicitly on adult family members’ perspectives and experiences. To enhance implementation, consideration of context is emphasized [16, 24], for which reason this study focuses on support in the intended setting of outpatient colorectal cancer (CRC) care.

Previous literature stresses the importance of tailoring support to meet the needs of individual family members [14, 25]. Tailored support is recognized as support co-created through an intangible interaction between the supporter and the supported [26]. How to offer implementable yet tailored support thus requires an understanding of both the perspectives of the supporter and the supported and how they relate. However, previous studies tend to focus on either the supporter, as in, healthcare professionals [27, 28] or the supported, as in, family members [29–31]. As a result, the co-created dimension is lost, and by that, also the opportunity to identify how support meets or fails to meet family members´ needs in a real-world setting. Such knowledge may guide the development of support models for family members that can be used in cancer care.

## Methods

This study aims to describe and contrast family members´ supportive care needs with support from CNs across the care process in outpatient CRC care. Therefore, the study is designed as a qualitative single case study with two embedded units to understand support for family members as it occurs in its natural setting [32–34]. For this study, the case is “perspectives on support for family members”, and the two embedded units are family members and CNs. Data is constituted by individual semi-structured telephone interviews with family members and CNs. Both within and across units, the analyses were undertaken using conventional qualitative content analysis as outlined by Hsieh and Shannon [35]. All decisions and uncertainties were documented and reporting adheres to the Consolidated Criteria for Reporting Qualitative Research checklist [36].

### Setting

We studied support within the CRC outpatient care process. The 16 clinics involved in this study complied with Swedish national guidelines for CRC care: The primary treatment is surgery, although complementary treatments (e.g., chemotherapy or radiation therapy) are sometimes also needed. For this study, the CRC care process is outlined in accordance with these guidelines and starts with a *diagnostic phase*, referring to the time between diagnosis and surgery, followed by a *treatment phase*, referring to the time from surgery until treatment is complete. Lastly, a s*urveillance* phase begins when the patient and family members are informed that treatment was successful, and no further treatment is expected and lasts until end the of the follow-up period.

The CNs are the coordinators of CRC care and are assigned to assess patients´ and family members´ supportive care needs. Further, they are assigned to provide support or refer those in need of support to a clinical social worker (CSW). Some clinics have CSWs available for family members, whereas other clinics refer family members to a primary healthcare center. The frequency and type of contact (physical meetings or telephone) with the patients differ between clinics. Face-to-face contact with family members primarily occurs at the time of diagnosis. All clinics have a locally defined description of the CN assignment, complementing national guidelines, although none clarify the meaning of support for family members. Each full-time employed CN is responsible for approximately 100 new patients diagnosed each year.

### Recruitment

To access information-rich cases [37], the participants were recruited purposefully. The inclusion criteria for the family members were being a family member of a person diagnosed with CRC and having the ability to read and understand Swedish. The inclusion criteria for CNs were a minimum of one year’s experience caring for persons diagnosed with CRC.

Family members were recruited from four outpatient CRC clinics via a nurse (not involved in the study). CNs were recruited from 16 different outpatient CRC clinics via the head of the departments. Presumptive participants were provided verbal and written information about the study by the gatekeepers. Those interested were contacted by MS and given time to ask questions. The interviews were scheduled at the participants’ convenience and written informed consent was obtained before the interviews were conducted.

### Data collection

Individual interviews were conducted from January to April 2021 (CNs) and from May to October 2022 (family members). The interviews followed interview guides (Additional file 1) based on research of family members’ experiences, supportive care needs, and supportive care. The interviews followed the CRC care process chronologically (diagnosis, treatment, surveillance), and the questions were open-ended and complemented with probing and prompting questions. To evaluate the interview guides, pilot interviews were undertaken with no corrections needed. To build rapport, all interviews started with casual conversation and collecting background data followed by the opening question, *Could you please tell me about when [your family member] was diagnosed with CRC*, or *Could you please tell me about your work as a CN?* The interviews were audio-recorded with the participants’ permission. The interviews with the family members ranged from 25 to 64 min (mean 53) and the interviews with CNs ranged from 28 to 63 min (mean 42).

### Analysis

Five interviews from each unit were transcribed verbatim by MS and the remaining by professional transcribers. All transcripts were checked for accuracy by MS. Following conventional qualitative content analysis [35], MS and AE first read the transcripts independently to become familiar with the data.

#### Within analysis

Sections of transcripts that were found to be related to the study aim were marked and given initial codes. ME and AE discussed and compared their initial codes and selected sections of transcripts. Each unit was coded separately. Examples of initial codes on the transcribed interviews with family members included *worries*, *emotions*, *good care*, *wanting to learn more practical things about the care*, and *no energy to take care of themselves*. For the CNs, examples of initial codes included *to involve the family members*, *patient in focus*, *body language*, and *availability*. The CRC care process was used as a timeline in the analysis.

#### Across units analysis

Coded sections were iteratively compared across units through discussions between MS and AE. The coded sections were grouped into subcategories, during which a main category was created. The categories’ characteristics were formulated by MS and KE, followed by continued analytical discussions between all authors until consensus.

#### Creating narratives

Narratives illustrating the respective perspectives of the family members and CNs were created by the researchers from the transcribed interviews. Creating narratives to reflect research participants’ perspectives on their experiences is a commonly used method in social sciences [38]. In accordance, we chose to illustrate the participants’ perspectives on support and supportive care needs through the story of a family member and a CN going through the CRC care process. All perspectives raised during the interviews are represented in these narratives, illustrated as the family members’ and CNs’ reflections. Last, MS returned to the transcripts to ensure the results derived from the data set and thatall relevant aspects had been incorporated into the analysis and narratives.

### Reflexivity

To prevent unintentional impact on research processes and results, it is important to reflect on personal and professional views of the phenomenon under study [37]. Three of the authors are registered nurses, and one is a registered CSW, which may have affected the research. To illuminate the authors’ pre-understandings and their potential implications for the research process and results, their thoughts on the phenomenon, along with uncertainties and decisions made during the research process, were documented and reflected upon throughout the study.

## Results

The participants comprised of 23 adult family members and 21 CNs. The family members were partners and adult children from across the cancer trajectory. Further characteristics are presented in Table 1.

**Table 1:** Participant characteristics (*n* = 44)

	Family members^1^ (*n* = 23)	Contact nurses (*n* = 21)
Age in years, mean (range)	57 (29–85)	52 (33–63)
Years experience as a registered nurse, mean (range)	-	22 (7–36)
Gender, n (%)		
Male	7 (30)	-
Female	16 (70)	21 (100)
Highest level of Education, n (%)		
Nine years’ compulsory school	1 (4)	-
Upper secondary school	6 (26)	-
Higher education	15 (65)	21 (100) *

^1^ Missing data: *n* = 1 education for family member

* All contact nurses (CNs) (*n* = 21) had a bachelor’s degree in nursing (120 ECTS - European Credit Transfer System). Among them, 7 CNs had additional formal CSN education recommended in national guidelines (7.5 ECTS), and 3 CNs had a master’s degree in oncology nursing (60 ECTS of university education)

**Fig. 1:**
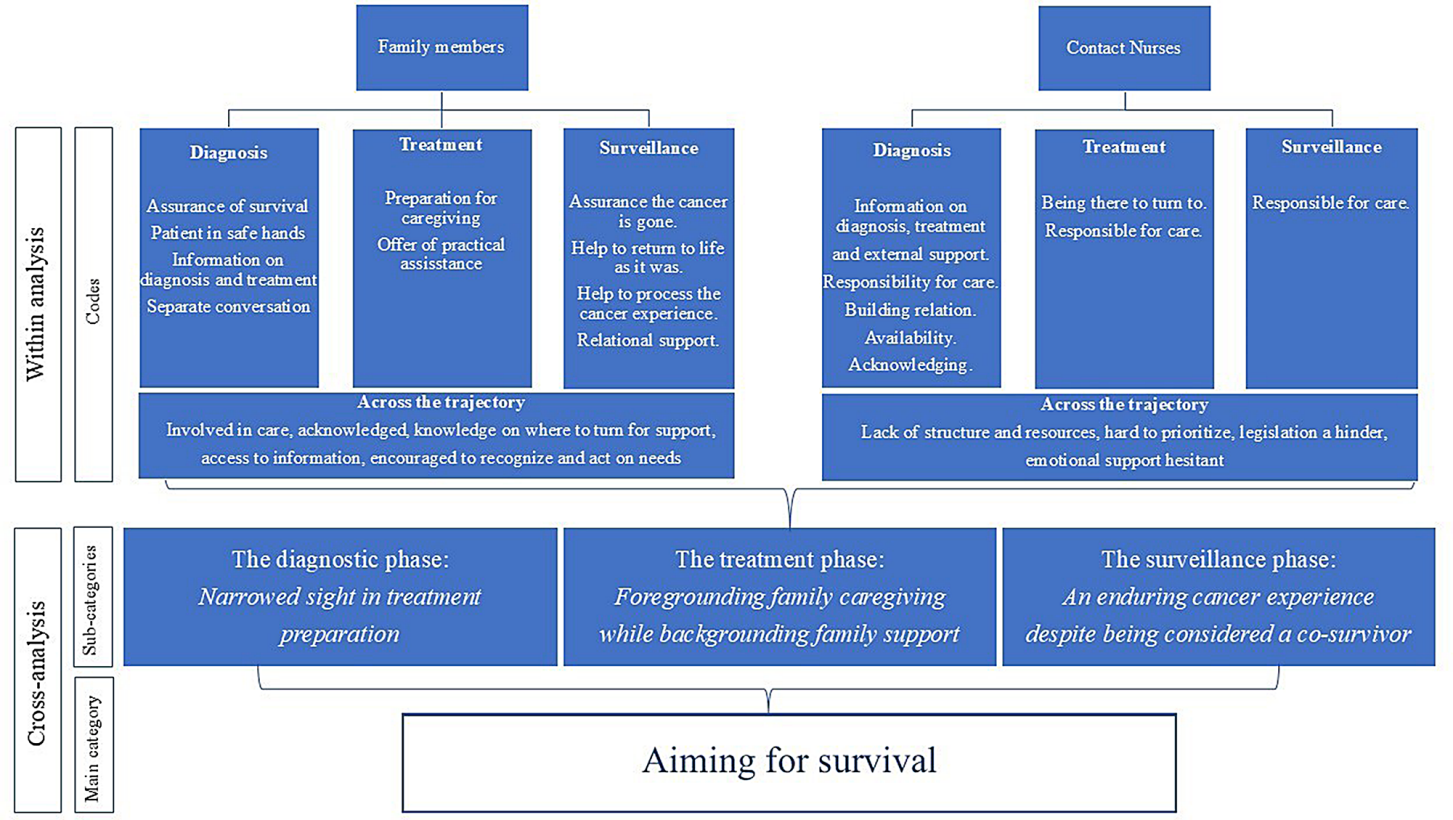
An illustration of the result of the analytical process

The result is presented following the CRC care process under the main category *Aiming for survival*, and three subcategories: (1) *The diagnostic phase: Narrowed sight in treatment preparation*, (2) *The treatment phase: Foregrounding family caregiving while backgrounding family support*, and (3) *The surveillance phase: An enduring cancer experience despite being considered a co-survivor.* The main category and subcategories are described in individual sections, with each subcategory further illustrated by narratives from the perspectives of family members and CNs, developed during the final step of the analysis (Fig. 1).

### Aiming for survival

Family members’ supportive care needs and support from the CNs were overshadowed by their common goal: the patients’ survival. This had the implication that, at the diagnostic phase, the perspectives of both family members and CNs was narrowed, primarily focusing on optimizing the patient for treatment. Similarly, during the treatment phase, the focus was on optimizing the recovery. The patient’s recovery was shouldered by family members, while support moved to the background, as the surgery had been completed. Given that survival was the goal, no planned contact was made between the CNs and the family members after the treatment and recovery was deemed successful, although for the family members, the cancer experience unexpectedly endured.

#### The diagnostic phase: Narrowed sight in treatment preparation

During this phase, both CNs and family members concentrated on optimizing the treatment outcome. The cancer prognosis directed attention toward the surgery. This phase was described by both the family members and CNs as well structured, with a strong emphasis on providing and receiving information about patient care. CNs supported the family members by allocating time for their questions, showing empathy, and informing them about the possibility of meeting with a CSW (which could include counseling). The family members reported that the provided information and being assured that the patient was in safe hands met their immediate needs, leading them to decline meetings with the CSW. However, the family members left the meeting with unmet needs as the shock of the cancer diagnosis hindered their ability to fully comprehend the information provided by the CNs. Further, the family members also reported feeling excluded from the care procedure due to the highly structured care (that they nevertheless appreciated). Additionally, the family members struggled to express existential concerns.

#### A narrative from the family members’ perspective

*We just found out that my dear wife has cancer. Unbelievable! Even though*,* in some way*,* we expected this*,* I still cannot believe this is happening. I was invited to join my wife to the appointment at the clinic*,* which felt good. I mean*,* we have shared a life together*,* of course we share challenging times as well. We met with a physician and a CN*,* who informed about the findings and what lay ahead. I really appreciated this meeting. They were compassionate and kind yet professional and competent. They informed us comprehensively and let us ask all the questions we had. Most importantly*,* we were told my wife would survive*,* the tumor was easy to remove*,* and they had a clear plan. What a relief! The CN offered us to meet with a CSW*,* which felt good but was not needed. I mean*,* she would survive! Instead*,* lets focus on getting there. They told us we didn´t have to engage in care*,* that they would take care of everything. This was comforting. I don’t think care should depend on family members. Yet*,* I mean*,* I wanted to be involved! For us*,* cancer treatment is a family project*,* just as all other projects we have been through together.*

*Despite being told that she will survive*,* I can’t stop my mind from wandering. I mean*,* it’s still cancer! What if it spreads? And the surgery and anesthesia are risky too*,* right? My wife isn’t that young anymore. If she dies*,* God forbid*,* I’m not sure if I could keep the house or manage everything. Oh*,* and how could we ever tell our children? They would be devastated… and our grandchildren too… Is this where our common life ends? I look at my wife. I could never*,* ever share these thoughts with her. I don’t want to upset her or burden her with my worries. I suppose it would be different if the prognosis were worse. Then we would need to have these conversations. So instead*,* I asked the CN about practical matters*,* like the time of the surgery*,* even though that wasn’t what was really on my mind.*

#### A narrative from the CNs’ perspective

*Today we informed a woman and her husband about a colon tumor. These meetings are always hard for the families. Still*,* we have a well-organized cancer care*,* really good surgeons*,* and I mean*,* the prognosis is good! But I think it’s a good thing that family members participate in these meetings. One can tell when the patient starts to wander in thoughts and stops listening. Then*,* family members can help to remember information. In addition*,* family members secure the patient at home and facilitate compliance with treatment. Informed family members definitely make the care process run smoother. While the physician informs about the diagnosis and treatment*,* I try to read their reactions. I know a cancer diagnosis are heavy news and that difficult times lay ahead. I try to comfort and provide emotional support through compassionate eye contact*,* also with the family members*,* to ensure that we see them as well. Further*,* we try to ease the burden for the family by assuring them that we take full responsibility for the care process. They can just follow our plan. For me as a CN*,* comforting the patient and family members by emphasizing availability is crucial. Since I know that there are no further scheduled meetings with the family members*,* I try to build a relationship with them and hope that this will make them contact me if needing support later on. In addition*,* I inform them about the possibility to meet with a CSW if needing emotional support.*

The narratives illustrate the gratitude the participating family members felt for their immediate needs being met, while also revealing lingering thoughts and suppressed concerns. Additionally, the narratives illustrate how focusing on the patient’s immediate needs and survival obscures the emotional impact this phase has on family members.

#### The treatment phase: Foregrounding family caregiving while backgrounding family support

During this phase, unlike the previous phase, family members felt overwhelmed with unexpected care responsibilities. They reported unmet needs for information, uncertainty, and a constant need to be on alert. From the CNs’ perspective, they had informed the families comprehensively at the time of the diagnosis and transferred care responsibilities to the surgical ward, expecting them to involve and prepare family members for the treatment phase. After surgery, the CNs described their role as providing background support and being available if needed. While some family members found this availability supportive, others felt the CNs were exclusively there for the patient. When family members did reach out, it was mainly for practical caregiving questions rather than to discuss their feelings or experiences. The CNs, however, preferred to discuss patient care directly with the patients. The family member’s overt focus on caregiving led the CNs to conclude that this reflected the family members’ needs during this phase.

#### A narrative from the family members’ perspective

*Finally*,* my wife is home from surgery. I’ve been so worried*,* and it felt like surgery took forever. Even though I’m glad that she’s home*,* my concerns aren’t over. Rather*,* the opposite. Last night was stressful*,* and she needed pain relief. I wonder how many pills she can take? And the injections*,* for how long? I remember they warned about infections and fever*,* but what exactly should I look out for*,* and where should we go if that happens? Fortunately*,* I can work from home. I could never leave her like this. Right now*,* I put everything else aside and focus exclusively on supporting her so that she’ll recover. She has difficulties leaving the bed and the stoma is leaking. I wasn’t expecting this. I try my best to help*,* but she wants to manage herself. I guess it’s kind of private. That’s why I feel I can’t share my concerns with common friends. And she doesn’t want to eat anything. Food must be a good thing for recovery*,* I think? Oh*,* I was so happy I could pick her up from the hospital*,* now I feel lost. I feel my wife’s care depends on me*,* but I don’t know how to do that? Until now*,* there were lots of contacts with healthcare professionals*,* and then it just stopped. As if we were done. We are certainly not done! I wish I had asked other questions when I had the chance*,* but I didn’t know what to ask. I have no prior experience with this*,* and they said I didn’t have to be involved. Oh*,* I don’t have the time or energy to search for where to turn to. I wish I had a CN to contact*,* as my wife has. Or maybe I can call her nurse. After all*,* my concerns are about her care.*

#### A narrative from the CNs’ perspective

*Yesterday*,* a worried husband contacted me. He was concerned about his wife’s appetite and wanted advice. These questions surprised me*,* since we inform clearly about this. In general*,* due to the extensive information we give initially*,* family members rarely contact us. If family members do contact us*,* it´s almost always about practical questions about patient recovery. I guess they don´t have further needs since the cancer has been removed. However*,* when it comes to patient care*,* I prefer to speak with the patient directly. It´s a question of integrity. And even though the family members mean well*,* sometimes they push the patient too much. Or too little*,* when it comes to mobilization. And sometimes they don’t agree with the patient´s decisions*,* and then I must take a stand for the patient. Yet*,* at times*,* when family members call several times about a certain matter*,* I can´t help thinking that perhaps they need something else? Like*,* some are really anxious. I wish we had a peer-support group to offer the family members. And sometimes I wish we could contact the family member for their own sake. But it wouldn´t be possible to do more within our care organization. And I mean*,* what if a family member feels really*,* really*,* low? What are my responsibilities then as a registered nurse? They don´t even have a medical record here.*

These narratives illustrate how the emotional toll from the narrow focus during the diagnostic phase carries over into the next phase. Failing to address the emotional impact of the diagnostic phase leaves family members feeling uncertain and overwhelmed in the treatment phase. Even though the patient has survived the surgery (what everyone was aiming for), the emotional and relational effects are becoming more evident.

#### The surveillance phase: An enduring cancer experience despite being considered a co-survivor

During this phase, family members were relieved that the patient had survived and moved past the intense treatment. However, contrary to their expectations, the cancer experience persisted. While they felt relief, happiness, and gratitude, concerns about recurrence and the shadow of death lingered. Some experienced changes in family dynamics, leading to frustration, loneliness, and a need for relational support. Previously overshadowed personal needs also emerged, causing confusion due to the expectation of only positive emotions. Consequently, there was a need to process the cancer experience. CNs recognized the intensity of the family members’ emotional experiences and their need to process these feelings, although contact between CNs and families diminished because the treatment phase had ended.

#### A narrative from the family members’ perspective

*We’re so relieved! Treatment has terminated*,* and we´ve been informed it looks good. Only a few weeks ago*,* I got really scared that the cancer was back*,* so I convinced my wife to call the hospital to assure us that the cancer is really gone. I hope it stays that way! One can never be sure once cancer has entered the body. Now*,* when it´s all over*,* the treatment and all*,* I start to realize what we have been through. At times*,* I feared our life together was over! It is like*,* now I can feel all feelings and fears that set aside for the sake of my wife’s treatment and everything. Now I can feel how intense and stressful this was. I thought I would be so happy once we reached here*,* which I am (!)*,* but I also feel sad*,* and sometimes*,* I cry for no obvious reason. I start to reconsider counseling to process my experiences. I think I would have said “Yes” if offered a counselor now*,* even though I said “No” previously. But then I didn’t know that I would feel like this. And during treatment*,* reaching out for help to find counseling for my own sake was unfeasible. It was all about helping my wife recover.*

*Survival was the goal we strove for. I imagined that we would go back to our active and social life*,* as it was before. We used to travel*,* we love to go for long walks*,* and we have lots of friends. But now that we’re here*,* my wife has no energy*,* and I feel her spark is gone. I try to encourage her to do things*,* maybe if she starts*,* she will regain her energy? But she just wants to stay at home. So now I go for our evening walks on my own. I can understand that the stoma is keeping her from seeing our friends*,* but I really miss doing activities together. I’ve tried to talk to her*,* but she keeps me out. It’s like something has come between us. But still*,* a counselor is for those really distressed right? I´m just a bit confused*,* for sure*,* it´ll be better soon? But I don’t know how to help my wife. I don’t know where to turn.*

#### A narrative on the CNs’ perspective

*The patient just underwent the one-year follow-up controls*,* and we were happy to inform them that everything looks fine. The next meeting is in two years. They seemed happy about this information. However*,* I got the feeling the husband had more concerns. He didn’t tell*,* it was just a feeling I got. Sometimes*,* I think that the family members’ own feelings catch up once the treatment is over. In general*,* I think that healthcare to a larger extent should care for patients and family members during this phase. But at the moment*,* this is not something that we can offer.*

The narratives illustrate how the emotional and relational effects of cancer care, continue to build up and become integrated into both family life and friendships. These effects not only influence the present moment and near future but may extend into the more distant future.

## Discussion

This study aimed to describe and compare family members´ supportive care needs with support from CNs across the care process in outpatient CRC care. The findings illustrate that the CNs support both meets and fails to meet the family members´ needs. Meeting with professional and empathic healthcare professionals who provide information about the diagnosis and treatment while assuring that the patient is in safe hands was perceived as supportive. In addition, being offered support was experienced as supportive per se. These findings corroborate previous research regarding the importance of both informational and emotional support [1, 14, 16, 30], the importance of support from the healthcare professionals caring for the patient [30, 39], and the importance of recognizing the family members’ situation. These are essential elements to incorporate when designing support for family members. Identified mismatches between support and supportive care needs will now be discussed in search for strategies in how to overcome them.

First, the main category, *Aiming for survival*, draws attention to the centeredness of somatic care and its implications for support and supportive care needs. The dominant patient focus, in which support for family members is primarily offered to improve patient outcomes, is reported in several studies [39–42]. As the family members´ supportive care needs only partly correspond with the CRC care process, the current centeredness seems insufficient to help family members maintain their health or prevent ill health from developing. A shift of centeredness is therefore warranted. In line with previous studies, we suggest workplace strategies at cancer clinics [43], educational interventions for CNs [44], and integrating family-centered viewpoints in undergraduate nursing education [45] as useful strategies. This family-centered viewpoint does not contradict the need for CNs’ to also focus on patient survival. Rather, it calls for a critical reflection on what “support for family members in outpatient cancer care” should entail, so that support can be better designed to benefit the whole family.

Second, there was a discrepancy between the timing of support and when the support was needed. The CNs’ support corresponded with the cancer treatment trajectory instead of the family members’ cancer experiences. Support was offered when the CNs and family members were fully focused on preparing the treatment, and when their individual needs were backgrounded. When the families’ supportive care needs surfaced in the second phase of the cancer trajectory, the support was perceived as inaccessible. While the CNs offered support at the timepoint recommended by international literature on supportive cancer care [20], it seemed insufficient in meeting the needs of family members. The analysis suggests that supportive care should be offered throughout the care process and also address lingering worries that may persist after the patient is considered free from cancer. A study on user-involvement [46] also highlight that the perceived relevance of support is closely linked to its timing. Additionally, the information provided at the time of diagnosis could be improved by better preparing family members for their cancer trajectory. Despite the CNs’ efforts to inform and guide family members through the treatment, several unmet needs arose due to perceived unexpected events and not adapting the information to the family members’ emotional responses. According to Edman et al. (2024), the CN may also need to regulate the emotional intensity during their meetings to ensure that family members have a better chance of being involved and not zoning out. Some of the families´ needs, such as those related to post-surgery caregiving or the realization that survival does not necessarily mean a return to pre-cancer life, could be seen as predictable by CNs, as they have been documented for decades and illustrate a gap between research and practice [47]. The findings suggest that CNs prepare family members for both caregiving and the common emotional responses that may linger well after the patient is cancer-free. This could also help families anticipate possible relational and social changes in everyday life, thereby easing some of their effects. Written and verbal information on where to find support throughout the entire care process and ensuring that this support is not overlooked due to stress responses and the initial focus on survival could also be incorporated.

Lastly, because family members avoided sharing their concerns to protect their loved ones, and because the CNs primarily addressed family members’ needs by asking if they had any questions while the patient was present - overlooking cues that indicated supportive needs - this led to unmet needs. As shown in a recent review [45] the family members’ behaviors, recognized as “protective buffering”, are common in families affected by cancer. Protective buffering means that both cancer patients and their family members protect each other by not voicing their concerns. Although this can be a coping strategy for some [48], it may hinder open communication and lead to isolation, loneliness [49], and decreased relationship functioning [50], as described by family members during the surveillance phase. Protective buffering is likely related to the well-known phenomenon of family members declining emotional support [51, 52], which was also observed in this study. These family members may be at risk for negative health outcomes even during years of survivorship [53, 54].

Thus, the results suggest that the perception of supportive care needs as being multidimensional, changing across the cancer trajectory, and connected to the individual [55], may incorporate “protective buffering” as an overarching element and serve as a basis for support initiatives. Supported by previous studies [56], we suggest that interventions should enhance family communication and ensure that each family member can express their concerns. This may include meetings where family members can speak to a CN or CSW alone, without worrying about the impact their concerns may have on the patient.

### Methodological considerations

This study used a Qualitative Case Study (QCS) design with embedded units to describe and compare family members´ supportive care needs with support from CNs across the care process in outpatient CRC care. QCS is a commonly used qualitative research method when the purpose is to obtain a deeper understanding of a phenomenon in a real-life context [57, 58]. Furthermore, QCS facilitates comparisons between the units in the case [59], which can deepen the understanding of the differences and similarities found in the data sets.

Although QCS is considered suitable for the study’s objectives, concerns have been raised about its rigor [34, 60], particularly regarding the difficulty in validating the research process. Efforts were therfore made to enhance rigor by incorporating reflexivity and addressing credibility, dependability, confirmability, and transferability [61]. For dependability, all analytical decisions and uncertainties were documented to provide a detailed audit trail, and both the interview guides and the analytical process are available for review. For credibility, all authors have experience in conducting qualitative research and MS conducted all the interviews, which were recorded and transcribed verbatim. The number of interviews was considered sufficient based on assessments of redundancy and the analysis was discussed between several authors until a consensus was reached. Telephone interviews have been criticized for providing less rich qualitative data than face-to-face interviews [62, 63]. However, they have been shown to produce equally rich qualitative data (ibid.). For this study, telephone interviews enabled the collection of data from a wide geographical area and meant less intrusion in the participants’ life.

Describing the context is important in QCS [34, 58]. Combining workplace interviews with observations in those settings could have potentially enriched the data set and enabled more detailed descriptions of the clinical context. That said, interviewing family members in their homes (made possible by phone calls), where the effects of the support were experienced, also provided valuable contextual insights. By presenting the setting through the voices of the participants, the study prioritized their perspectives of what takes place. Through presenting the setting and the participants’ characteristics, the readers may experience transferability to similar contexts and care processes. Furthermore, the transferability is deemed enhanced by the geographic variation of the included participants.

Further research may include both interviews and observations in clinical and domestic contexts, where the experiences of the CNs’ support are manifested. Evaluating suggested strategies to enhance family support in cancer care is also needed. This study also highlights the need to clarify the legal responsibility of the CNs connected to the family members’ health. Additionally, future research may evaluate the potential benefits of CSW taking on an outreach role for family members. Further research may also complement this study by exploring how tailored support can be offered to family members < 18 years old.

## Conclusion

From interviews with family members and CNs, areas where support meets supportive care needs, and areas where needs are left unmet were identified. Professional and empathetic healthcare professionals who provide information about diagnosis and treatment, along with assurances of a good prognosis and that the patient is in safe hands, offer valuable support. However, unmet needs emerged throughout the cancer trajectory. To effectively support family members, support must be based on their cancer experience and established knowledge about their supportive care needs. Family members require preparation for and support during their entire cancer trajectory. In addition, they need guidance on where and who to turn to at each stage. This could possibly be achieved by strengthening the collaboration between CNs and CSWs. CSWs could introduce themselves to family members in the same way as other health professionals, serving as a consistent point of contact throughout the cancer journey. Additionally, strategies for assessing needs and developing support should consider protective buffering.

## Electronic supplementary material

Below is the link to the electronic supplementary material.

Supplementary Material 1

## Data Availability

The datasets used and/or analyzed during the current study are available from the corresponding author on reasonable request.

## Abbreviations

CN: Contact Nurse
CRC: Colorectal cancer
CSW: Clinical Social Worker
QCS: Qualitative case study
