# Supplementary material for: Aiming for survival: a qualitative single case study of support for family members across the care process in outpatient colorectal cancer care
Source: BMC Cancer. 2025 May 12;25:856. doi: 10.1186/s12885-025-14245-2 (PMC12067705; doi:10.1186/s12885-025-14245-2)
Supplement: Supplementary file 1 — Supplementary Material 1 [file 12885_2025_14245_MOESM1_ESM.pdf]

Additional file 1. Interview guides.

Opening question FAMILY MEMBERS	Opening question CONTACT NURSE
Please tell me about what your life has been like during this period.<br>*(Following the cancer trajectory chronologically)*	*Please tell me about your work as a CN.*<br>*(Contact with family members following the cancer trajectory chronologically)*
**Support** (Emotional and spiritual support, information and practicalities)<br>• Existing<br>• Desired<br>• Changed since earlier phases<br>• The coming phases/ the future	Perception of support to family members
**Needs** (Emotional and spiritual needs, information and practicalities)<br>• Personal<br>• Those of other family members<br>• Changed since earlier phases<br>• The coming phases/ the future	Family members' perceived needs
**Resources/ Hinders**<br>• Personal<br>• External<br>• Changed since earlier phases<br>• The coming phases/ the future	Resources / hindrances for support
**Concluding questions**: If you were to guide a family member *who is about to enter your situation*, what would you have wanted to communicate to this person?<br>"What would you have liked the support for family members to look like?	**Concluding question:** *In a perfect world with endless resources, how would you design the support to family members of persons with colorectal cancer?*
